# Clinico-Haematological Profile of Acute Megakaryoblastic Leukaemia: Report of Five Cases

**DOI:** 10.1155/2009/461912

**Published:** 2008-01-28

**Authors:** Sunita Sharma, Anita Nangia, Sonal Jain Malhotra, Shashi Narayan, Aparna Harbhajanka, Sarika Singh

**Affiliations:** Department of Pathology, Lady Hardinge Medical College, New Delhi 110001, India

## Abstract

Acute megakaryoblastic leukemia (AMKL) is a rare subtype of acute myeloid leukemia. Although known as a distinct entity for a very long time, because of lack of distinct clinical features and morphological criteria, it is difficult to diagnose this variant correctly. We herein present the clinical, morphological, cytochemical, and immunocytochemical features of five cases of AMKL. Certain morphological features such as presence of abnormal platelet count, giant platelets, and cytoplasmic blebbing in blasts were found to be important pointers towards the diagnosis. However, none of the features were found to be consistent and thus morphological diagnosis has to be confirmed by cytochemistry and immunocytochemistry.

## 1. Introduction

Acute megakaryoblastic leukemia (AMKL) is a rare entity and accounts for 3–5% of all acute myeloid leukemia [1]. Although described in literature for more than 70 years [2], the clinical profile and morphological criteria for its diagnosis remain ill defined. It can be confused with acute lymphoblastic leukemia ALL-L1/acute myeloid leukemia AML-M0. It may arise de novo or may be secondary to chemotherapy, or progress from myeloproliferative neoplasm (MPN) and/or myelodysplastic syndrome [1, 3–5]. It has a bimodal peak of distribution (in adults and children 1-2 years of age). Children with Down's syndrome have a particularly higher incidence of AMKL [6]. Here we describe five cases of AMKL with variable clinicohaematological presentations and discuss the diagnostic difficulties faced in arriving at a definitive diagnosis.

## 2. Materials and Methods

All acute megakaryoblastic leukemias diagnosed in the Department of Pathology of Lady Hardinge Medical College, New Delhi, in the period 1997 to 2007, were included in the study. A total of five cases were retrieved. The clinical history and other details were taken from the case files.

Wright's stained peripheral smears and bone marrow aspirate (BMA) smears were examined. Bone marrow biopsy was available in only one case.

Cytochemistry, including myeloperoxidase (MPO), Sudan black B (SBB), periodic acid Schiff (PAS), and nonspecific esterase (NSE), was available in all the cases.

Immunophenotyping was done in 4 out of 5 cases. In 3/4 cases, immunocytochemistry was done by APAAP technique using DAKO antibodies (Figure 3) and in the fourth case flow cytometry results were available.

## 3. Results

Clinical and haematological profiles of our 5 cases are summarized in Tables 1  and 2.

Most of the cases were males (M:F = 4:1). Out of the 5 cases, two were adults while the other three were in the pediatric age group (1.5 years to 6 years).

Four out of the five cases had an acute onset illness and presented with symptoms of marrow infiltration while the fifth patient had a long history of 3 years duration which is described separately.

One patient out of these 4 cases had features suggestive of Down's syndrome (Case IV). Hepatosplenomegaly and lymphadenopathy were observed in two and one patient, respectively (Table 1).

All the four cases were anemic. Leucocytosis was noted in 3 while one case showed thrombocytosis (Case II). Giant platelets were present in Case I only.

**Figure 1:**
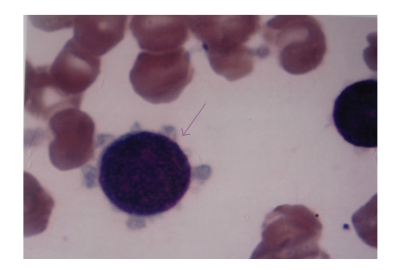
Megakaryoblast with cytoplasmic blebbing (1000X).

**Figure 2:**
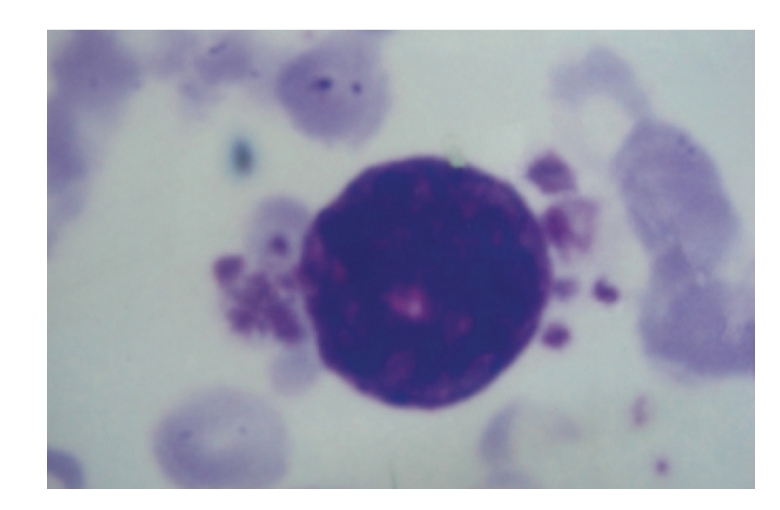
Megakaryoblast with platelet budding (1000X).

**Figure 3:**
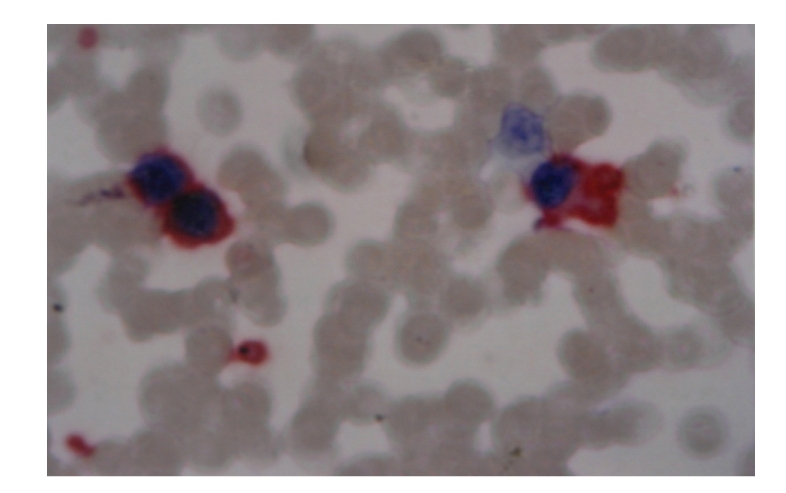
CD61 positivity in blast (200X).

Peripheral blood blast count was in the range of 4% to 40%. The blasts were large, 3-4 times the size of a small mature lymphocyte with moderate to abundant agranular basophilic cytoplasm. The nuclei were round to oval with fine to stippled chromatin & 1-2 prominent nucleoli. Few blasts in all the cases showed cytoplasmic blebbing (Figure 1). Platelet budding was observed in 3/4 cases (Figure 2).

Bone marrow aspirate smears were cellular in three out of four cases showing blasts ranging from 25% to 56% of all nucleated cells with morphology similar to that seen in the peripheral blood along with presence of micromegakaryocytes and promegakaryocytes. In one of the cases, blasts showed clustering mimicking metastases (Case I). In the fourth case (Case IV), repeated attempts of BMA yielded diluted marrow. Bone marrow biopsy in this case showed blasts with clustering and increased reticulin fibers (Grade III).

The fifth case (Case V) had a long duration illness and presented with weakness and abdominal distension for 3 years.

This patient had massive splenomegaly, 7 cm below costal margin and mild hepatomegaly. The patient was anemic with a normal total leukocyte count and platelet count. Peripheral smear showed a leukoerythroblastic blood picture with presence of tear drop cells and 25% blasts. Bone marrow aspirate revealed 28% blasts. These blasts had morphology and cytochemical findings similar to those of the other cases (Table 2). Based on these findings, a diagnosis of acute megakaryoblastic leukemia arising as acute transformation of myeloproliferative neoplasm (primary myelofibrosis) was made. Bone marrow biopsy and immunophenotyping was suggested. However, the patient left against medical advice and was lost to follow up.

## 4. Discussion

AMKL is a rare leukemia, accounting for 7–10% of childhood AML [7] and 1-2% of adult AML [8, 9] and confers a poor prognosis. Patients with Down's syndrome have an increased incidence of AMKL and have a good prognosis [6, 10]. AMKL can arise de novo or as a secondary event post chemotherapy or progress from myeloproliferative neoplasm or myelodysplastic syndrome [1, 3–5]. In our study, one patient was possibly secondary to MPN (primary myelofibrosis).

It is difficult to diagnose this variant solely on the basis of morphology. However, there are features like clustering of blasts, presence of cytoplasmic blebbing, and platelet budding which may be useful in clinching the diagnosis [7]. In our study these features were present in majority of the cases thus emphasizing their importance. Cytochemistry is not very helpful although necessary to rule out other leukemias. Bone marrow fibrosis if present is a very important feature and is seen in a high proportion of cases [6]. In a study conducted at M. D. Anderson Cancer Centre including 37 cases of AMKL, bone marrow fibrosis was present in 62% of the cases [11], although in our study, bone marrow biopsy was available in only one case which showed fibrosis. Immunophenotyping is necessary to confirm the diagnosis which could be performed in 4 out of the 5 cases.

Thrombocytosis was present in one patient who was a 22-year-old female while the other adult patient had a normal platelet count. All the three pediatric patients were thrombocytopenic. This observation is similar to that seen in other studies [12], thus emphasizing the fact that AMKL can be categorized as either undifferentiated or differentiated, with more differentiated ones occurring in adults [13].

## 5. Conclusion

AMKL is a rare leukemia. It is important to correctly diagnose this variant in view of its prognostic implications. Although immunophenotyping is the gold standard, it is not available in all the centers. Careful search for features like cytoplasmic blebbing, platelet budding, bone marrow fibrosis, clustering of blasts, and cytochemical positivity for nonspecific esterase which is fluoride resistant can help in correctly diagnosing a significant number of AMKL.

## Figures and Tables

**Table 1:** Clinical features and hematological parameters.

Features	Case I	Case II	Case III	Case IV	Case V
Age/sex	6 years/M	20 years/F	1.5 years/M	2 years/M	22 years/M
C/F	Pallor, easy bruisability	Pallor, dyspnea, gum bleeds, malena, hematemesis	Fever, petechiae	Fever, epistaxis, seizures	Fever
Down's syndrome	—	—	—	+	—
Hepatomegaly	+	—	+	—	+
Splenomegaly	+	—	+	—	+
Lymphadenopathy	—	—	+	—	—
Hb (g/dL)	10.7	4.9	4.8	5.1	9.4
WBC (×10^9^/L)	40	30	17.5	7.1	7.9
Plt count (×10^9^/L)	30	480	14	60	203

**Table 2:** Morphology, cytochemistry and immunocytochemistry.

		Case I	Case II	Case III	Case IV	Case V
Morphology						

Peripheral smear	*Red cells					
	Normocytic Normochromic	Present	Present	Present	Present	Present with tear drop cells
	*Blasts (%)	18%	40%	4%	5%	25%
	*Blast morphology					
	(i) agranular blue cytoplasm with cytoplasmic blebbing	Present	Present	Present	Present (some blasts were granular)	Present
	(ii) Giant platelets	Present	Absent	Absent	Absent	Present
	(iii) Platelet budding	Present	Present	Absent	Absent	Present

Bone marrow aspiration/biopsy	*Hypercellular marrow showing megakaryoblasts, micromegakaryocytes, promegakaryocytes (full range of differentiation)	Present with clustering mimicking metastasis	Present	Present	Diluted BMA & Biopsy showed blasts with clustering and increased reticulin fibres	Present
	*Blast (%)	25%	45%	56%	25% (biopsy)	28%

Cytochemistry						

	PAS	Positive	Positive	Negative	Positive	Positive
	NSE	Positive, fluoride resistant	Positive, fluoride resistant	Positive, fluoride resistant	Positive, fluoride resistant	Positive, fluoride resistant
	MPO/SBB	Negative	Negative	Negative	Negative	Negative

Immunocytochemistry						

	Lymphoid markers (CD5, 7,19, 20)	Negative	Negative	Negative	Negative	Negative
	Myeloid markers (CD13,33)	Negative	Negative	Negative	Negative	Equivocal
	CD61 (Gp Illa)	Positive	Positive	Positive	Positive	ND
